# Case report: Anti-NMDA receptor encephalitis manifesting as rapid weight loss and abnormal movement disorders with alternating unilateral ptosis and contralateral limb tremor

**DOI:** 10.3389/fimmu.2022.971514

**Published:** 2022-09-15

**Authors:** Yue Han, Sizhu Gong, Yafei Wan, Xiyao Fu, Enling He, Min Liu, Fang Deng

**Affiliations:** Department of Neurology, The First Hospital of Jilin University, Changchun, China

**Keywords:** autoimmune encephalitis, anti-NMDA receptor encephalitis, limb tremor, unilateral ptosis, rapid weight loss, topiramate

## Abstract

Anti-N-methyl-D-aspartate receptor (NMDAR) encephalitis, associated with immunoglobulin G (IgG) autoantibodies against the GluN1 subunit of the NMDAR, is one of the most common types of autoimmune encephalitis. In patients with anti-NMDAR encephalitis, movement disorders (MDs) are often frequent, mainly presenting as facial dyskinesias and stereotyped movements. The alternating clinical manifestation of limb tremor with unilateral ptosis is rare. Here, we report an interesting case of a 22-year-old woman with rapid weight loss presenting with staged dyskinesia. Interestingly, she typically showed persistent tremor of the right upper limb, which would stop when her left upper eyelid drooped uncontrollably, a phenomenon that lasted for a few seconds, followed by automatic upper eyelid lift and continued persistent tremor of the upper limb. Moreover, it was fortunate to find anti-NMDAR antibodies in her cerebrospinal fluid (CSF), which indicated the patient had anti-NMDAR encephalitis. And abnormal apparent diffusion coefficient (ADC) hyperintense signals on the left midbrain interpeduncular fossa explained this manifestation of focal neurological deficit. After the systematic administration of immunotherapy (intravenous immunoglobulin, IVIG), steroid pulse therapy, and symptomatic treatment, the initial symptoms were significantly relieved except for limb tremor. The MDs were becoming less visible for the next six months under topiramate prescriptions. Noteworthy, there are no specific MD phenotypes in anti-NMDAR encephalitis. We describe the young women with unique MDs and rapid weight loss to help us get a more comprehensive understanding of anti-NMDAR encephalitis.

## Introduction

Anti-N-methyl-D-aspartate receptor (NMDAR) encephalitis is a neuroinflammatory disease mediated by autoantibodies against NMDARs, first described in 2007 (1). The NMDAR is glutamate-gated calcium channel comprised of two GluN1 and two GluN2 or GluN3 subunits (2, 3). In these affected patients, the NR1 subunit of the NMDARs is attacked by antibodies leading to the capping and internalization of surface NMDARs and the reversible loss of NMDAR-related synaptic function resulting in neurological and psychiatric manifestations (4–8).

The clinical manifestations are characterized by psychiatric symptoms, seizures, cognitive impairment, abnormal movements, disturbances of consciousness, autonomic dysfunction and insufficiency of ventilation, mainly affecting young women, with or without associated tumors, and with teratomas as the most common associated tumors (4, 5, 9). During the course of the disease, approximately 75% of adults will develop MDs without a specific dyskinesia phenotype, often manifesting as facial dyskinesia and stereotyped movements (10, 11).

Now, there is no uniform standard for the diagnosis of anti-NMDAR encephalitis. Among relevant clinical tests, the detection of NMDA antibodies in CSF or serum is the gold standard for diagnosis, whereas the brain magnetic resonance imaging (MRI) findings lack specific features (5) and electroencephalogram (EEG) shows typical extreme delta brush only in only one-third cases (12).

To early identify this encephalitis in terms of clinical symptoms, we describe the first case of atypical anti-NMDAR encephalitis presenting with alternating unilateral limb tremors and contralateral ptosis with rapid weight loss.

## Case report

In late February 2021, a 22-year-old female was admitted to the hospital for persistent right upper limb tremor with paroxysmal left ptosis. In the past five months, the patient took two job-hunting exams and was in a nervous state. During the period, she lost about 30 kg of weight from 80 kg (BMI = 30.5 kg/m^2^) to 52 kg (BMI = 19.8 kg/m^2^). Three months before admission, even after finding a satisfactory job, her weight still dropped rapidly, along with the loss of appetite and insomnia. She did not deliberately diet and exercise to lose weight and had no history of drug ingestion, psychiatric disorder, infection, and vaccination. On January 25, 2021 (1 month before admission), her right upper limb first developed involuntary, rhythmic, and slight tremors, which appeared at rest, worsened while moving casually or feeling nervous, and disappeared during sleeping. On admission, the amplitude of persistent tremors in the right upper limb increased (**Supplementary Video 1**), and an interesting phenomenon of paroxysmal left ptosis emerged. At this point, ptosis and limb tremor did not affect each other. Her physical and neurological examination revealed only a fluctuating left oculomotor nerve palsy. When the left upper eyelid began to droop involuntarily, her left eyeball moved outward and downward, and she showed limited adduction of the left eye, diplopia, and slurred speech. When her left eye opened again, the strange symptom lasting a few seconds would completely disappear. And the remaining neurological examination results, including other cranial nerves, muscle power, muscle tone, tendon reflexes, sensory nervous system, and Babinski signs, were not observed to be abnormal. The 14-item Hamilton Anxiety Scale (HAMA-14) and 17-item Hamilton Depression Scale (HAMD-17) score, respectively, was 13 (<7 is no anxiety, more than 7 is possible anxiety, more than 14 is definitely anxiety, more than 21 is obvious anxiety, and more than 29 is very severe anxiety) and 16 (<7 is no depression, 7~17 is possible depression, 17~24 is definitely depression, and more than 24 is very severe depression), suggestive of mild anxiety and depression states.

**Figure 1:**
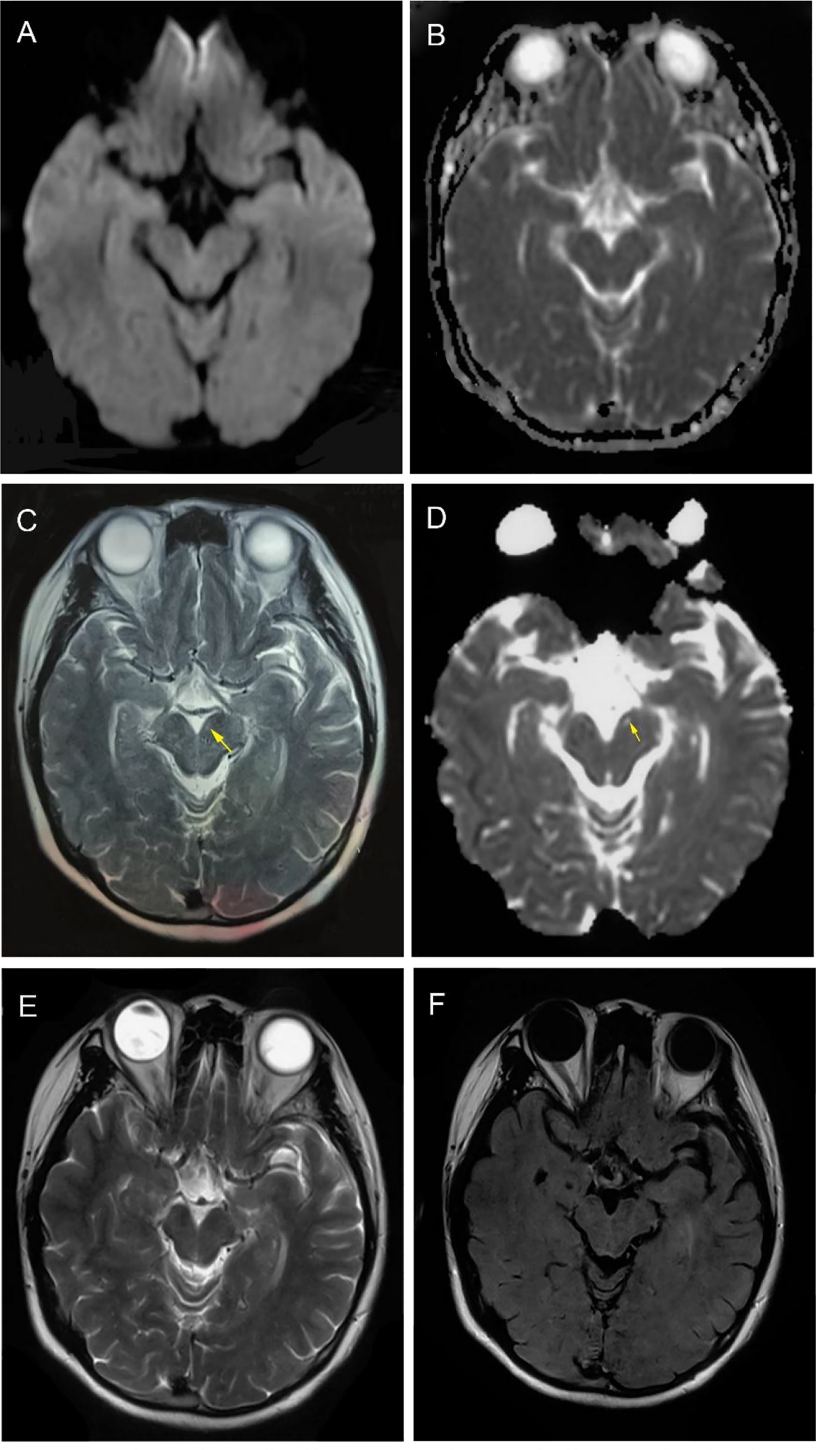
Magnetic resonance imaging findings. At time of diagnosis, brain MRI DWI and ADC sequences (transverse) showed no suspicious signal alterations **(A, B)**. Four months after onset, T2 and ADC sequences showed obvious high signals on the left midbrain interpeduncular fossa (highlighted by arrows) **(C, D)**. One year later, T2 and T2-flair sequences (transverse) showed midbrain lesions disappeared. **(E, F)**.

At first, trihexyphenidyl was administrated to relieve the abnormal tremor. Besides, brain MRI showed no apparent abnormalities (**Figures 1A, B** **)**. 24h video electroencephalogram revealed sharp waves and slow spike waves were emitted from both temporal sides during waking and sleeping stages. And when the ptosis occurred, the corresponding EEG did not show abnormal rhythmic changes. In laboratory investigations, we did not get a lot of positive information. Her thyroid function test showed increases in anti-thyroid peroxidase antibodies (TPO-Ab) (92.86IU/mL) [normal range: 0~5.61] and anti-thyroglobulin (A-Tg) (13.95IU/mL) [normal range: 0~4.11], while fT3, fT4, and TSH were in normal rang. Serum folate levels (2.3 ng/mL) [normal range: 3.1~20] decreased and homocysteine levels (18.49 umol/L) [normal range: 0~15] increased. Regarding liver and renal function, there were mild protein and urea decrease and moderate uric acid elevation. Other tests for metabolic and infectious encephalopathies were within the normal range: full blood count, erythrocyte sedimentation rate, lactate, β-hydroxybutyric acid, urinary ketone bodies, C-reactive protein, serum rheumatoid factor (RF), anti-streptolysin O, immunoglobulin G (IgG), IgM and IgA, complement 3 and 4, antineutrophil cytoplasmic antibody (ANCA), antinuclear antibodies (ANAs), antidoublestranded DNA, anticardiolipin antibody (ACA), cyclic citrullinated peptide (CCP), and infection markers (HBsAg, HBsAb, HBeAg, HBeAb, HBcAb, HCVAb, HCVcAg, HIVAb, HIVAg, and TPAb). Moreover, serum tumor markers (β-HCG, HE-4, CYFRA21-1, CEA, CA242, CA125, NSE, AFP, SCC, CA199, and CA153), ultrasounds of the reproductive system, and whole-body fluorodeoxyglucose-PET (FDG-PET) imaging assessment showed no evidence of metastatic tumors. After six days of symptomatic treatment, the patient’s clinical status did not improve. Given the patient’s clinical manifestations and abnormal metabolic indicators, we tested blood copper, blood ceruloplasmin, and corneal K-F ring. These normal results helped us to rule out the possibility of hepatolenticular degeneration (HLC).

However, lumbar puncture results indicated a new direction for a definite diagnosis and further treatment. CSF test was clear and colourless with a pressure of 140 mmH_2_O [normal range: 80-180], mild IgG increase (69.69 mg/L) [normal range: 0-34], normal leucocytes level (6×10^6/L) [normal range: 0-8×10^6], normal protein level (0.33 g/L) [normal range: 0.15-0.45], normal glucose level (3.39 mmol/L) [normal range: 2.3-4.1]. CSF cytologic examination revealed occasional lymphocytes and no abnormal cells and further excluded the presence of neoplastic elements. Surprisingly, the cell-based assay (CBA) test found that the anti-NMDAR antibody was negative in the serum [normal range: <1:10] but positive in the CSF [titer 1:1++, normal range: <1:1]. The results were the same by checking again. Additionally, it is worth mentioning that the initial dilution gradients of serum and CSF are 1:10 and 1:1, respectively. And the number of “+” represents the fluorescence intensity at that dilution gradient. Each dilution gradient corresponds to 1 to 3 “+”. The more “+” means the higher the antibody content at the dilution gradient. The cut off of serum and CSF for positive is 1:10 and 1:1 on the NMDAR test in our laboratory. Besides, IgG of other autoimmune encephalitis antibodies (LGI1, CASPR2, GABA_B_R, AMPAR1, AMPAR2, IgLON5, DPPX, GAD65, mGluR5, GlyR, and D2R), IgG of central nervous system (CNS) demyelinating antibodies (AQP4, MOG, MBP, and GFAP), IgG of anti-neuronal antibody (Hu, Yo, Ri, CV2 (CRMP5), Amphiphysin, Ma1, Ma2, SOX1, Tr (DNER), Zic4, GAD65, PKCγ, Recoverin, and Titin (MGT30)), and IgG and IgM of ganglioside antibodies (Sulfatide, GM1, GM2, GM3, GM4, GD1a, GD1b, GD2, GD3, GT1a, GT1b, and GQ1b) in serum and CSF were all negative.

Based on the presence of specific anti-NMDAR antibodies and related clinical judgment results, the patient was eventually diagnosed with anti-NMDAR encephalitis on the seventh-day post-admission and given a 5-day course of IVIG treatment (22.5 g/d, 0.4 g/kg). On the third day of IVIG administration, a new strange clinical manifestation of alternating left-sided ptosis and right-sided upper limb tremor occurred (**Supplementary Video 2**). She usually presented with a persistent tremor of the right upper limb. But sometimes, the left upper eyelid would involuntarily droop, after which the tremor of the right upper limb would stop and last for about a few seconds. Then the left upper eyelid lifted automatically, immediately followed by the restart of persistent tremor in the right upper limb. At this point, ptosis and limb tremor affected each other. Similarly, when the left upper eyelid began to droop involuntarily, her left eyeball moved outward, and she showed double vision and could not speak clearly. Twelve days after hospitalization, high-dose intravenous methylprednisolone pulse therapy (1000 mg/d for 3 days, 500 mg/d for 3 days, 240 mg/d for 3 days, 120 mg/d for 3 days) was given immediately following the IVIG. Then oral methylprednone tablets was at an initial dose of 48 mg daily with a slow tapering schedule of 4 mg every week. Then we replaced trihexyphenidyl with topiramate capsules to control the tremor. With high-dose steroidal therapy, the patient’s symptoms were significantly improved compared with before. The frequency of left ptosis was reduced considerably, and the right limb shaking could also occasionally stop for a few seconds at the break. When she left the hospital (27 days post-admission), paroxysmal involuntary ptosis of the left upper eyelid wholly disappeared, but the tremor was still not entirely under control. By then, her weight had dropped to 48 KG (BMI = 18.3 kg/m^2^).

Two weeks after the discharge, the tremor aggravated again, gradually affecting the head and left upper limb. The manifestations gradually alleviated after increasing the dosage of topiramate. Two months later, the patient’s conditions steadily improved, her weight increased to 55 Kg (BMI = 21.0 kg/m^2^) with a good appetite, and only a slight tremor of the right upper limb remained. T2 and ADC showed abnormal high signals on the left midbrain interpeduncular fossa (**Figures 1C, D** **)**. The MRI findings of the patient were not common in the literature (4, 5, 13). During the one-year follow-up, the patient’s tremor has been completely controlled without any sign of recurrence and tumor (**Supplementary Video 3**). In July 2022, a repeated brain MRI showed midbrain lesions disappeared. (**Figures 1E, F** **)**.

## Diagnosis and differential diagnosis

The young woman presented with abnormal movement disorders with anxiety and insomnia, consistent with the primary symptom of anti-NMDAR encephalitis. Moreover, NMDAR antibodies were detected in CSF but not in serum with CBA, which was not surprising because NMDAR antibodies were intrathecal synthetic antibodies, and their testing was more sensitive in CSF than serum. Besides, combined with the patient’s unusual clinical manifestation, weight loss, and abnormal signals in the midbrain on MRI, we suspected the possibility of epileptic disorders, mitochondrial diseases, HLC, CNS malignancy, demyelination, and even vasculitis. However, the corresponding EEG did not show abnormal rhythmic changes when the ptosis occurred. And lactate, blood copper, blood ceruloplasmin, and corneal K-F ring were negative. Tumor markers, FDG-PET, and repeated gynecological ultrasound examination showed no evidence of metastatic tumors. We suggested that she should have a gynecological ultrasound examination every six months. CNS demyelinating antibodies and rheumatological and immunological indicators such as ANCA were negative. Therefore, the possibility of these above diseases can be excluded. In short, the patient met the diagnostic criteria for NMDAR encephalitis according to Graus et al. (14) and was finally diagnosed with anti-NMDAR encephalitis.

## Discussion

In slightly more than ten years, anti-NMDAR encephalitis has become the most frequently recognized neuronal-antibody-mediated encephalitis with a variety of clinical manifestions (6). Both MDs and neuropsychiatric manifestations are core clinical features of this encephalitis. In general, patients exhibit more than a kind of abnormal movements, such as prominent stereotypes, dystonia, and chorea, with an almost complete absence of tremors (10, 15, 16). There were many previous findings of unique and variable MDs in anti-NMDAR encephalitis. Dubey et al. reported a patient with bilateral thalamic lesions resulting in hemichorea and dystonia (17). Rossi et al. found a patient with episodic ataxia as the main manifestation (18). Recently, a case mimicking NMDAR encephalitis was reported in which the patient presented with bilateral ptosis and external ophthalmoplegia and MDs of the face and limbs, but these manifestations were persistent and occurred simultaneously (19). In addition, a few cases were accompanied by limb tremors, but none were specific (20, 21).

**Figure 2:**
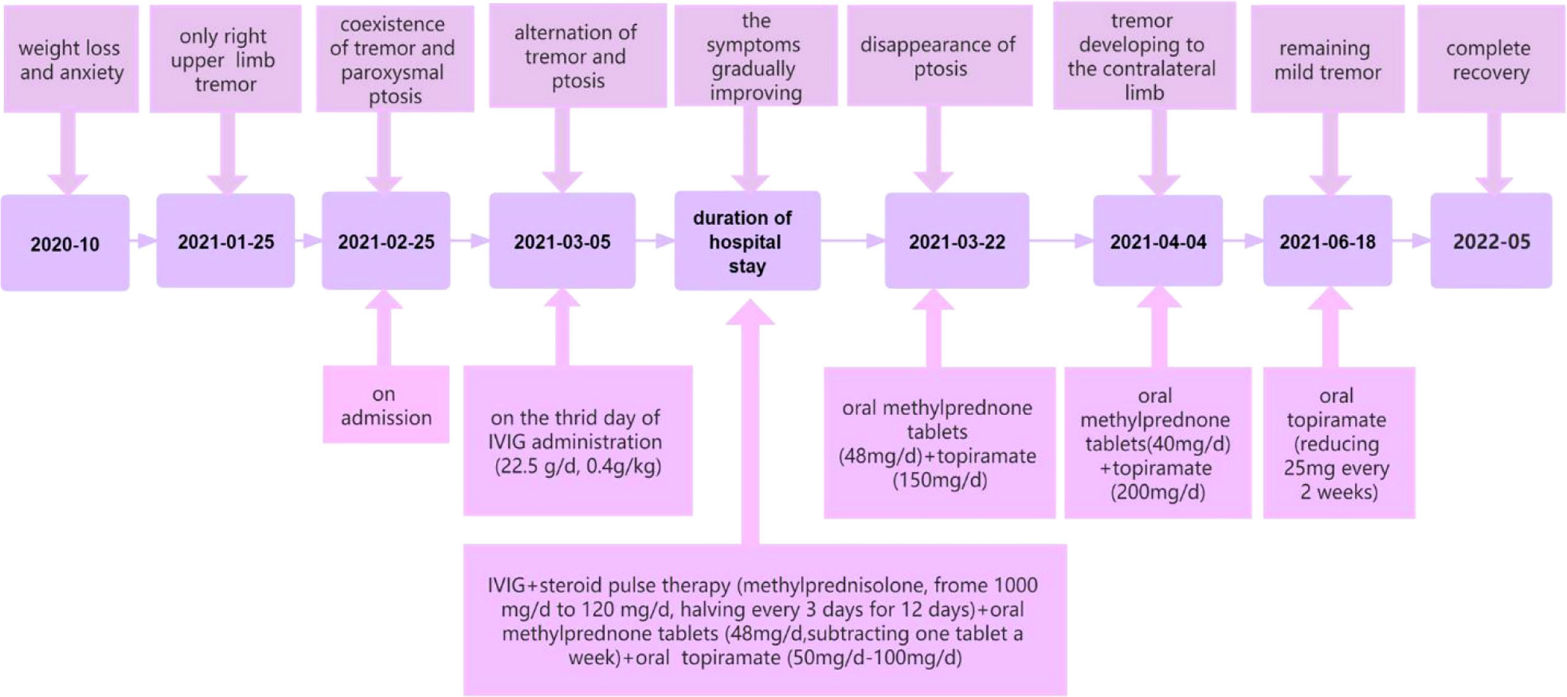
Related symptoms and treatment at different time points.

To our knowledge, we have presented a particular case with anti-NMDAR encephalitis. Unlike other reported patients in the literature, this patient presented with alternating left-sided ptosis and right-sided upper limb tremor during the disease. As mentioned above, her abnormal movements were divided into six main stages. Tremor only, the coexistence of tremor and paroxysmal ptosis, alternation of tremor and ptosis, the disappearance of ptosis, progression of tremor to the contralateral side, and remaining mild tremor (**Figure 2**). In addition, the patient had definite and significant weight loss and insomnia before and after onset. We speculate that the reasons why the patient’s symptoms are so atypical and unique are the attack of low titer antibodies on NMDAR, the effect of drug therapy on disease progression, and the role of NMDARs in food intake.

### The attack of low titer antibodies on NMDAR

It has been proved experimentally that anti-NMDAR antibodies are pathogenic (5). Besides, antibody titers in CSF and serum are related to the clinical outcome (4, 5). We believe that antibody-mediated glutamate receptor blockade is an acceptable assumption for the abnormal motility in this patient (22).

According to drug research, this hypothesis has been confirmed in humans and animals. In human beings, non-competitive antagonists of NMDARs such as phencyclidine and ketamine induce repetitive orofacial and limb movements (5, 23, 24). And in rodents, locomotor and stereotypical behaviors can be seen with drugs that antagonize NMDAR function (25).

Targeted antibody-mediated cross-linking and internalization of NMDARs leads to decreased synaptic NMDARs density and alteration of synaptic plasticity (5, 6, 26, 27), occurring in both inhibitory and excitatory neurons (27). Inhibitory GABAergic neurons contain high levels of NMDARs which are higher concentrations of NMDARs than other neuronal subtypes (5). Thus, antibody-mediated reduction of NMDARs predominantly leads to the inactivation of gabaergic neurons and increases in extracellular glutamate. The alteration of function of GABAergic inhibitory interneurons might lead to disinhibition of excitatory pathways. The homeostatic downregulation of inhibitory synapses on excitatory neurons could potentially enhance neuronal excitability and spontaneous discharges (15, 22, 23, 28, 29).

Hence, we assume that alternating limb tremors and ptosis might be caused by increased neuronal spontaneous discharges on the left midbrain interpeduncular fossa. The involuntary discharges in the area can stimulate the fibber tracts to the red nucleus and induce holmes tremor (HT) in the right limb (30), which is characterized by a combination of resting, postural, and intention tremor (30, 31). When the intensity of abnormal discharge is too strong and the range is too extensive, this conduction pathway and the ipsilateral oculomotor nerve can be damaged, manifesting in ipsilateral ptosis and cessation of right limb tremor.

In addition, now there is a widely accepted mechanism. Central pattern generators are neural circuits that produce the patterns of neural activity and central commands, specifically controlling stereotyped and rhythmic motor behaviors (32, 33). Among them, brainstem pattern generators are inhibited and controlled by GABAergic neurons (22). The decrease of NMDARs in GABAergic neurons can lead to the disinhibition of brainstem central pattern generators, releasing primitive limb movement and causing complex elaborate movements and dyskinesias (22, 23).

In this case, tremor improved significantly after topiramate treatment, which also proved the reliability of the above hypothesis. Because topiramate can be used to control tremors by potentiating gamma-aminobutyricacid inhibitory activity and blocking the excitatory neurotransmission of glutamate (34). So far, there has been a practical case of topiramate in treating HT (31). In another multicenter, placebo-controlled clinical trial of 208 people, topiramate was influential in treating moderate-to-severe idiopathic tremors (35).

As an autoimmune disease, anti-NMDA encephalitis is usually treated with first-line therapy (steroids, IVIG, and/or plasma exchange) and second-line therapy (rituximab or cyclophosphamide) could be considered if the response is inadequate (11, 36). In the treatment of MDs, choreoathetosis and ballismus are usually treated with dopamine 2 (D2) antagonists. However, for isolated dystonia, reduction of D2 antagonists and initiation of levodopa is necessary (36). In a case report, volatile anesthetic, especially isoflurane, was an effective way to control dyskinesia in anti-NMDAR encephalitis (37). We have given topiramate for the first time in anti-NMDAR encephalitis and achieved favorable results and prognosis, providing a novel guide for future treatment options.

### The effect of drug therapy on disease progression

This abnormal clinical presentation can be reversed by removing antibodies through immunotherapy (4). The multiple clinical features of the syndrome might be caused by a progressive decline of antibody-mediated NMDAR clusters and function in the early stages, followed by antibody removal and a gradual recovery of receptor function during recovery (23). Thus, multistage abnormal movements are not only related to the development of the disease itself but also to the elimination of antibodies during the course of taking the medication.

### The role of NMDARs in food intake

Several animal studies have shown that NMDARs are physiological mediators of eating behavior, and blocking these receptors can inhibit natural feeding and reduce weight (38).

The role of NMDARs in human eating behavior was systematically described for the first time through two reported cases of anti-NMDAR encephalitis associated with pathological eating behavior (39, 40). It states that eating disorders can be explained by the interaction of NMDAR-related neuroendocrine and reward systems in central and peripheral structures (39). In the neuroendocrine/homeostatic mechanisms, orexin is one of three critical biological factors in the control of feeding (39). Orexin A depending on NMDARs, can increase appetite, meal frequency, and length of meals (41). Antagonism by NMDAR antibodies can inhibit the action of orexin, thereby contributing to a decrease in appetite and food intake (39). This mechanism reveals the reason for the loss of appetite in our patients.

## Conclusion

This case highlights the importance of recognizing MDs and unexplained weight loss of anti-NMDAR encephalitis because it may be connected with alternating limb tremors and ptosis or other rare manifestations, which are often ignored.

On the one hand, the MDs are a common and notable feature of anti-NMDAR encephalitis, yet until now has unpredictable performance (15). On the other hand, weight loss caused by anorexia behavior is rarely reported in the anti-NMDAR encephalitis. When we are faced with those variable and impalpable MDs or rapid weight loss, considering the possibility of anti-NMDAR encephalitis is necessary.

Thus, we conclude the MDs of anti-NMDAR encephalitis are very heterogeneous, and the patients perhaps might be accompanied by a rapid decrease in weight. Meanwhile, topiramate might be a potential drug for the treatment of MDs in anti-NMDAR encephalitis. More importantly, CSF anti-NMDAR antibody test is crucial to recognize atypical autoimmune encephalopathy and take early immunotherapy.

## Patient perspective

When the strange symptoms appeared, I was very terrified. But when I received treatment, the tremor and ptosis gradually disappeared, and I began to gain confidence. Now, my symptoms have fully returned to normal, and I am happy to share my funny story.

## Data availability statement

The original contributions presented in the study are included in the article/**Supplementary Material**. Further inquiries can be directed to the corresponding author.

## Ethics statement

The patient in this case report provided written informed consent for publication.

## Author contributions

YH was involved in the diagnosis and treatment of the disease, collected the data and wrote the manuscript. SG performed conception and design. YW, XF, EH, and ML reviewed and revised the manuscript. FD supervised the review and approved the final version of the manuscript. All authors have read the final manuscript and approved it for submission.

## Funding

This work was supported by National Natural Science Foundation of China (grant number: 82071293).

## Acknowledgments

The authors thank the patient for sample contribution.

## Conflict of interest

The authors declare that the research was conducted in the absence of any commercial or financial relationships that could be construed as a potential conflict of interest.

## Publisher’s note

All claims expressed in this article are solely those of the authors and do not necessarily represent those of their affiliated organizations, or those of the publisher, the editors and the reviewers. Any product that may be evaluated in this article, or claim that may be made by its manufacturer, is not guaranteed or endorsed by the publisher.

## Supplementary material

Supplementary Video 1 Tremor only.

Supplementary Video 2 Alternating unilateral ptosis and contralateral limb tremor.

Supplementary Video 3 Complete recovery.
